# Loss of PTDSS1 in tumor cells improves immunogenicity and response to anti–PD-1 therapy

**DOI:** 10.1126/sciadv.adx8134

**Published:** 2025-09-10

**Authors:** Jielin Liu, Shelley Herbrich, Sreyashi Basu, Yulong Chen, Ashwat Nagarajan, Swetha Anandhan, Sangeeta Goswami, Liangwen Xiong, Baoxiang Guan, Padmanee Sharma

**Affiliations:** ^1^ Department of Genitourinary Medical Oncology, The University of Texas MD Anderson Cancer Center, Houston, TX 77030, USA.; ^2^ Graduate School of Biomedical Sciences, University of Texas MD Anderson UTHealth Houston, Houston, TX 77030, USA.; ^3^ James P. Allison Institute, The University of Texas MD Anderson Cancer Center, Houston, TX 77030, USA.; ^4^ Immunotherapy Platform, The University of Texas MD Anderson Cancer Center, Houston, TX 77030, USA.; ^5^ Independent Researcher, San Francisco, California, USA.; ^6^ Department of Immunology, The University of Texas MD Anderson Cancer Center, Houston, TX 77030, USA.

## Abstract

*PTDSS1* (phosphatidylserine synthase 1) encodes an enzyme that facilitates production of phosphatidylserine (PS), which mediates a global immunosuppressive signal. Here, based on in vivo CRISPR screen, we identified PTDSS1 as a target to improve anti–PD-1 therapy. Depletion of *Ptdss1* in tumor cells increased expression of interferon-γ (IFN-γ)–regulated genes, including *B2m*, *Cxcl9*, *Cxcl10*, and *Stat1*, even in the absence of IFN-γ stimulation in vitro. Loss of *Ptdss1* in tumor cells also led to increased expression of MHC-I, enhanced cytotoxicity of CD8^+^ T cells, and increased frequency of an iNOS^+^ myeloid subset. A gene signature derived from the iNOS^+^ myeloid cell subset correlated with clinical benefit in patients treated with anti–PD-1 therapy. Moreover, genetic and pharmacological inhibition of *Ptdss1* in different tumor models improved anti–PD-1 therapy. Together, our results provide insights on a therapeutic strategy for overcoming immunosuppression by inhibiting PTDSS1 and provide rationale for development of a combination immunotherapy strategy composed of PTDSS1 inhibition plus PD-1 blockade.

## Teaser

PTDSS1 is a therapeutic target to enhance tumor immunogenicity and improve antitumor responses with immune checkpoint therapy.

## INTRODUCTION

Immune checkpoint therapy (ICT) targeting proteins such as cytotoxic T lymphocyte–associated protein 4 (CTLA-4), programmed cell death protein 1 (PD-1), or programmed death-ligand 1 (PD-L1) has been transformative in many cancer types; providing durable clinical responses and markedly improved overall survival in a subset of patients (*1*–*3*). However, many individuals fail to respond or develop resistance to ICT (*2*, *4*), highlighting an urgent need for novel therapeutic combination strategies to improve ICT efficacy.

Numerous tumor-intrinsic factors have been implicated to contribute to primary and acquired resistance to ICT (*5*–*9*), including but not limited to the following: antigen presentation deficiency, caused by inactivating mutations in genes of the antigen presentation machinery (*10*, *11*); defective interferon-γ (IFN-γ) responses, caused by mutations in *STAT/JAK* pathway (*12*, *13*); and oncogenic pathway activation which impair intratumoral immune cell function, including phosphatase and tensin homolog (PTEN), wingless-related integration site (WNT)/β-catenin, epidermal growth factor receptor, and v-Ki-ras2 Kirsten rat sarcoma viral oncogene homolog (KRAS) (*14*–*16*).

In this study, we used an in vivo CRISPR-Cas9 knockout screen in a murine cancer model to identify phosphatidylserine (PS) synthase 1 (*Ptdss1*) as an additional intrinsic regulator of tumor cell fitness under anti–PD-1–selective pressure. *PTDSS1* encodes phosphatidyl serine synthase 1, which converts phosphatidylcholine to PS. PS, when localized in the cytoplasmic leaflet, serves as docking sites important for activation of multiple signaling pathways including RAS, AKT, and protein kinase C. Externalized PS can be recognized by PS receptors on phagocytes, leading to clearance of the stressed or apoptotic cells. Strategies targeting the immunosuppressive PS in the tumor microenvironment (TME) have shown promising results in preclinical studies but the clinical outcomes have been inconclusive (*17*–*19*). Recent studies suggest that PTDSS1 may modulate TME through mechanisms independent of surface PS exposure (*20*, *21*). Using genomic and pharmacologic inhibition of *Ptdss1*, we identified both tumor-intrinsic and tumor-extrinsic roles of *Ptdss1* in regulating response to ICT. Specifically, *Ptdss1* deficiency increased tumor cell response to IFN-γ, immunogenicity, and sensitivity to CD8^+^ T cell–mediated killing. *Ptdss1* deficiency in tumor cells also favored the development of a pro-inflammatory TME marked by an increase in cytotoxic CD8^+^ T cells. Moreover, *Ptdss1* deficiency skewed macrophages polarization toward a pro-inflammatory state in vitro and correlated with an increased frequency of iNOS^+^ myeloid cells in vivo. A gene signature derived from these iNOS^+^ myeloid cells correlated with improved response to ICT in two different patient cohorts. Preclinical studies in two murine models of solid tumors showed that loss of *Ptdss1* in tumor cells and pharmacologic inhibition of *Ptdss1* worked synergistically with anti–PD-1 treatment for improved antitumor response and survival. Our findings highlight a key role for *Ptdss1* in regulating tumor cell immunogenicity and shaping TME, providing a strong rationale for combining *PTDSS1* inhibition with PD-1 blockade to improve ICT efficacy.

## RESULTS

### In vivo CRISPR screen identifies known and potential regulators of immune checkpoint response

**Fig. 1.**
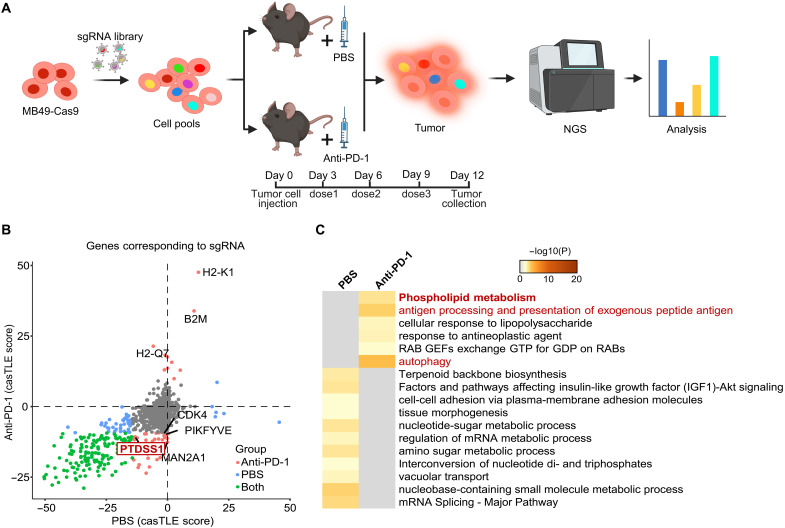
In vivo CRISPR screen identifies known and potential regulators of anti–PD-1 response. (**A**) In vivo CRISPR screen scheme. Created in BioRender. Liu, J. (2025) https://BioRender.com/5ak7l4w. (**B**) sgRNA composition changes in the PBS treated or anti–PD-1–treated tumors compared to that of cell library. Statistical analysis was done with casTLE software. Significantly enriched or depleted sgRNAs (*P* < 0.01) are labeled in color. Confidence in enrichment or depletion is indicated by casTLE score. (**C**) Functional enrichment analysis of the significantly changed sgRNAs in both anti–PD-1 and PBS condition.

To systematically explore potential targets in tumor cells that may improve the efficacy of anti–PD-1 therapy, we performed an in vivo CRISPR knockout screen using the MB49 tumor cell line which responds poorly to ICT (*22*–*24*) (Fig. 1A). We engineered a Cas9-expressing MB49 cell line (fig. S1A) and transduced the cells with sgRNA libraries targeting 2013 genes encoding kinases, phosphatases, and drug targets as well as 1123 nontargeting guides (*25*). After full representation of the sgRNA library in the cells was confirmed (fig. S1B), we transplanted the cell pools subcutaneously into C57BL/6 mice followed by treatment of three doses of anti–PD-1 or phosphate-buffered saline (PBS). Consistent with previous report, anti–PD-1 was able to delay tumor growth, but the effect was limited (fig. S1C) (*23*). Next, tumors from both groups were collected on day 12, followed by genomic DNA isolation and next-generation sequencing to quantify changes in library representation. CRISPR screen performance was validated as suggested by the differential distribution of a list of essential genes (*26*) compared to nonessential genes in all groups (fig. S1D). As we are interested in identifying genes that may regulate tumor cells response to anti–PD-1 therapy, sgRNA library composition of the tumors was first compared to that of the original cell pools and then plotted against each other (Fig. 1B). We identified a list of 48 sgRNAs showing enrichment or depletion specifically in the anti–PD-1–treated condition (table S1), suggesting that perturbation of the corresponding genes may regulate tumor cells fitness under anti–PD-1–selective pressure. Inspection of the gene list revealed well-known genes essential for immunotherapy, including components of the antigen presentation machinery *B2m*, *H2-k1*, and *H2-q7* (*11*, *27*). On the contrary, sgRNAs targeting *Cdk4*, *Man2a1*, and *Pikfyve* were significantly depleted in the anti–PD-1–treated group, consistent with the recent findings that genetic and pharmacological inhibition of these targets synergize with immune checkpoint blockades (*28*–*31*). We next sought to understand how the rest of the gene targets are involved in mediating anti–PD-1 response. To do so, we performed functional enrichment analysis using genes targeted by the 48 sgRNAs and included genes targeted by the differentially enriched sgRNAs in the PBS group as a reference. As expected, antigen processing and presentation pathway was enriched in the anti–PD-1–treated group (Fig. 1C). Autophagy pathway was also enriched in the anti–PD-1–treated group, which supports the increasingly recognized idea that autophagy is a conserved process used by tumor cell to evade immune surveillance and combination of autophagy inhibition with ICT is a promising strategy to overcome resistance to ICT (*31*–*36*).

### Inhibition of Ptdss1 synergizes with anti–PD-1 treatment

**Fig. 2.**
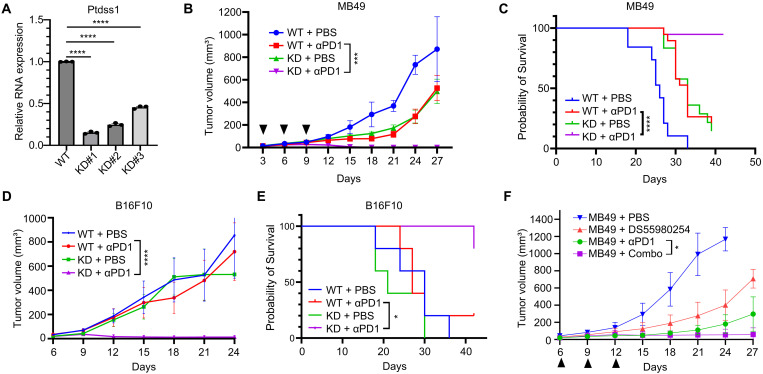
Loss of Ptdss1 sensitizes tumors to anti–PD-1 treatment. (**A**) RNA expression level of *Ptdss1* in WT or KD clones. (**B**) Representative tumor growth curve of mice transplanted with vector control MB49 cells (WT) or *Ptdss1^KD^* MB49 cells treated with PBS or anti–PD-1 (*n* = 9 to 10 for each group, experiments repeated three times; black arrow indicates treatment time point. (**C**) Representative Kaplan-Meier survival curves of mice transplanted with KD and WT MB49 tumors. (**D**) Tumor growth curve of WT or *Ptdss1* ^*KD*^ B16F10 tumors treated with PBS or anti–PD-1 (*n* = 5 for each group). (**E**) Representative Kaplan-Meier survival curves of mice transplanted with KD or WT B16F10 tumors. (**F**) Tumor growth curve of MB49 tumors treated with PBS, anti–PD-1, DS55982054, or combination of anti–PD-1 and DS55982054 (*n* = 10 for each group). Data are presented as the means ± SEM. *P < 0.05, ***P < 0.001, and ****P < 0.0001. Significance was determined by two-tailed unpaired Student’s *t* test for (A); two-way ANOVA for [(B), (D), and (F)]; and log-rank test for [(C) and (E)].

In addition to the autophagy and antigen processing/presentation pathways, phospholipid metabolism pathway was also enriched in the anti–PD-1–treated group. A key gene in the phospholipid metabolism is *Ptdss1*, which has been implicated to modulate tumor immune response (*20*, *21*). We found that knocking down *Ptdss1* (Fig. 2A), which encodes PTDSS1, significantly inhibited tumor growth when combined with anti–PD-1 treatment (Fig. 2B and fig. S2, A, B). Mice inoculated with knockdown (KD) tumor had significantly improved survival when treated with anti–PD-1 in both MB49 bladder cancer model and B16F10 melanoma model (Fig. 2, C to E). Moreover, pharmacological inhibition of PTDSS1 with DS55980254 (*20*), a recently developed PTDSS1 inhibitor, also improved anti–PD-1 efficacy (Fig. 2F). To determine whether loss of Ptdss1 led to any differences in cell proliferation and/or survival, we measured the cell growth rates in vitro by IncuCyte proliferation assay and in vivo by measuring tumor growth in immune-deficient NSG mice. Results showed that the cell growth rates of KD clones and wild-type (WT) cells were indistinguishable from each other (fig. S2C), and the tumor growth curves in NSG mice are comparable between KD tumors and control tumors (fig. S2D). These data are in line with the report that *Ptdss1* knockout mice were viable and fertile compared to their littermate control (*37*). Together, these data suggest knocking down *Ptdss1* in tumor cells sensitized tumors to anti–PD-1 treatment and this synergistic effect depends on an intact tumor immune microenvironment.

### Ptdss1 deficiency increases tumor cell immunogenicity and response to IFN-γ

**Fig. 3.**
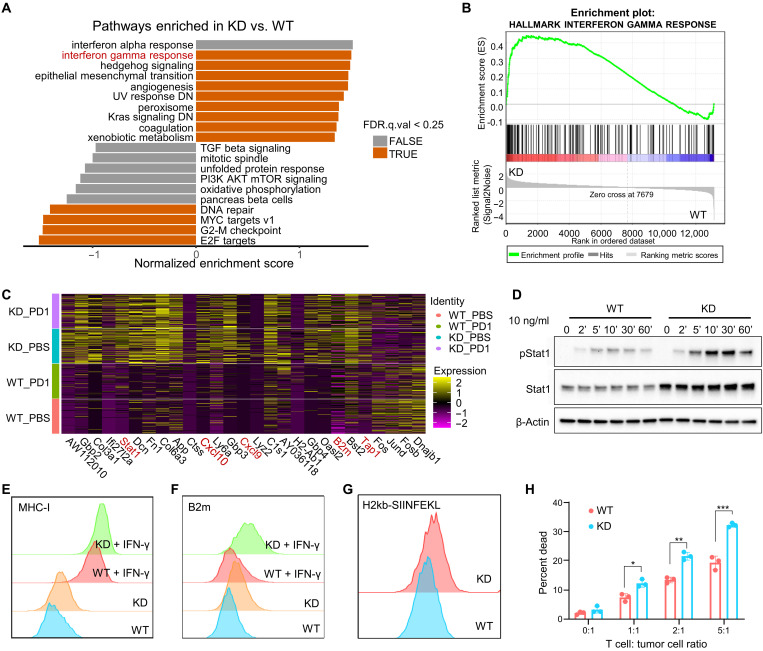
Loss of Ptdss1 increases tumor cell’s IFN-γ response and antigen presentation. (**A** and **B**) Gene set enrichment analysis (GSEA) results showing gene sets significantly enriched in KD or WT cells. NOM.p.value <0.05 was used to determine significance. Positive normalized enrichment score (NES) indicates enrichment in KD. (**C**) Differentially expressed genes in KD tumor cells compared with WT. Each column represents a single cell. Differentially expressed genes are defined as fold change ≥2 and *P* < 0.05. (**D**) Western blot results of control and KD cells treated with IFN-γ (10 ng/ml) for indicated times. (**E** and **F**) FACS results of surface MHC-I and B2M levels on KD and control cells treated with or without IFN-γ for 24 hours. (**G**) FACS results of surface H2kb-SIINFEKL level on IFN-γ treated KD and control cells pulsed with SIINFEKL for 6 hours. (**H**) FACS results of cell death of control or KD cells cocultured with OT-I CD8^+^ T cells at different ratios. Data are presented as the means ± SEM. *P < 0.05, **P < 0.01, and ***P < 0.001. Significance was determined by two-tailed unpaired Student’s *t* test for (H).

To understand how knocking down *Ptdss1* improved anti–PD-1 response, we first compared the transcriptomes of WT and KD MB49 cells cultured in vitro by RNA sequencing. Gene set enrichment analysis (GSEA) of the transcriptomes revealed that IFN-γ response pathway was enriched in the KD cells (Fig. 3, A and B). Loss of *Ptdss1* up-regulated expression of multiple IFN-regulated genes including *B2m*, *Cxcl9*, *Cxcl10*, *Stat1*, and *Tap1* even in the absence of exogenous IFN-γ (Fig. 3C). We also observed elevated basal level of signal transducers and activators of transcription 1 (STAT1), the major signal transducer of IFN-γ response pathway, and its phosphorylation when stimulated with IFN-γ (Fig. 3D). Expression levels of two downstream targets of IFN-γ response pathway, MHC-I and B2m, were also elevated in the KD cells, with or without IFN-γ stimulation (Fig. 3, E and F), suggesting an increased immunogenicity of the KD cells. When pulsed with ovalbumin (OVA) peptide SIINFEKL, KD cells exhibited higher level of H2kb bound SIINFEKL, which translated into increased sensitivity to OT-I CD8^+^ T cell–mediated killing (Fig. 3, G and H).

### Loss of PTDSS1 shapes an inflammatory TME

**Fig. 4.**
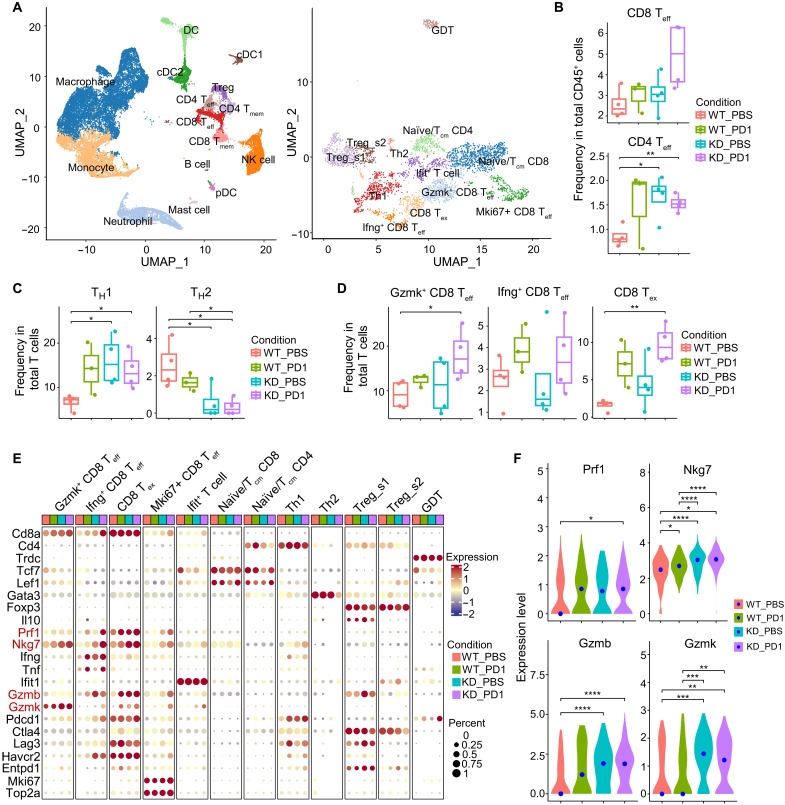
Loss of Ptdss1 in tumor cells induced an inflammatory TME. (**A**) tSNE plot of the cell clusters in total CD45 cells and total T cell identified by scRNAseq. (**B**) Frequencies of CD8 and CD4 T effector cell clusters in total CD45 cells. (**C**) Frequencies of T_H_1 and T_H_2 CD4 T cell cluster in total T cells. (**D**) Frequency of T_ex_, *Ifng* ^+^ CD8^+^ T_eff_, and *Gzmk* ^+^ CD8^+^ T_eff_ clusters in total T cells. (**E**) Dotplot showing expression level of T cell related genes in different T cell clusters. (**F**) Violin plot showing expression level of cytotoxic function related genes in cells in the *Gzmk* ^+^ CD8^+^ T_eff_ cluster. *P < 0.05, **P < 0.01, ***P < 0.001, and ****P < 0.0001. Significance was determined by two-tailed unpaired Student’s *t* test.

Successful antitumor response depends on an intricate interplay between tumor cells and their microenvironment (*7*, *38*). To investigate whether tumor-extrinsic factors contribute to the improved anti–PD-1 response in *Ptdss1* KD tumors, we transplanted KD or WT cells in mice, treated the mice with anti–PD-1 or PBS 3- and 6-days postinoculation, and profiled the immune subsets within the TME on day 9 by single-cell RNA sequencing (scRNAseq). On the basis of expression of cell type–specific marker genes, the immune cells were clustered into high abundance of different myeloid clusters including monocyte and macrophage, type 1 and type 2 conventional dendritic cells, neutrophils and relatively low abundance of CD4^+^, CD8^+^ T cells, and NK cells (Fig. 4A). As PD-1 is known as a T cell checkpoint receptor, we first looked at the lymphoid compartment of the TME. Consistent with previous reports from our group and others (*23*, *39*), anti–PD-1 treatment increased levels of CD8^+^ and CD4^+^ T effector cells (T_eff_) in WT tumors (Fig. 4B). Intratumoral level of CD4^+^ T cells, specifically the T helper cell 1 (T_H_1) subset, was also increased in KD tumors, regardless of treatment condition. In contrast, level of T_H_2 CD4^+^ cells was relatively lower in KD tumors compared to WT tumors (Fig. 4C). While anti–PD-1 treatment induced infiltration of exhausted CD8^+^ T cell (T_ex_), IFN-γ expressing effector CD8^+^ T cell (*Ifng* ^+^ CD8^+^ T_eff_) and Gzmk expressing effector CD8^+^ T (*Gzmk* ^+^ CD8^+^ T_eff_), we observed that loss of *Ptdss1* in tumor cells induced a further increase in frequency of *Gzmk* ^+^ CD8^+^ T_eff_ cells (Fig. 4D). Expression levels of genes related to cytotoxicity, including *Prf1*, *Nkg7*, *Gzmb*, and *Gzmk*, were also highest in CD8^+^ T cells in KD tumors treated with anti–PD-1 (Fig. 4, E and F).

**Fig. 5.**
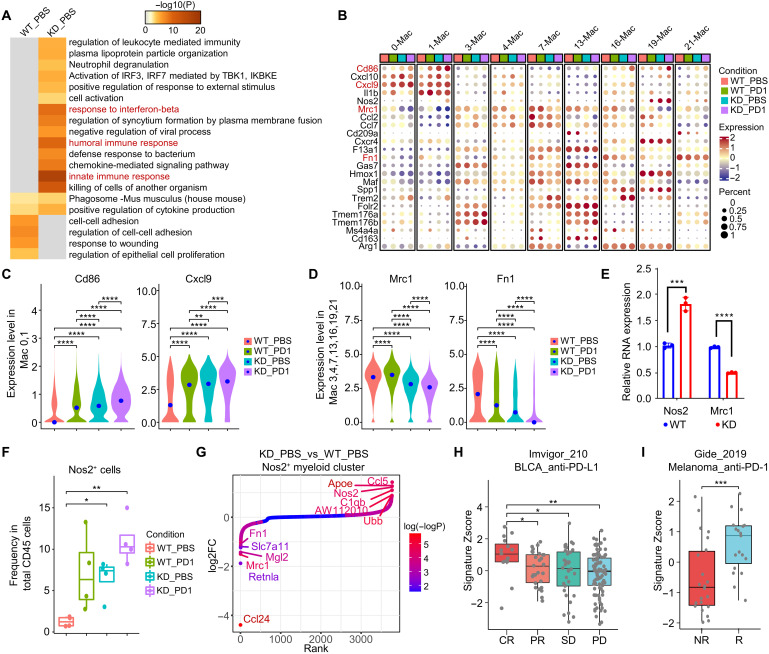
Loss of Ptdss1 in tumor cells increased the inflammatory potential of myeloid cells. (**A**) Functional enrichment analysis of the differentially expressed genes in macrophage cluster identified by scRNAseq in PBS treated WT or KD tumors. Differentially expressed genes are defined as fold change ≥0.5 and *P* < 0.05. (**B**) Dotplot showing expression level of macrophage related genes in different macrophage clusters identified by scRNAseq. (**C**) Expression of *Cd86* and *Cxcl9* in cells in the 0- and 1-macrophage clusters. (**D**) Expression of *Mrc1* and *Fn1* in cells in the 3-, 4-, 7-, 13-, and 16-macrophage clusters. (**E**) RNA expression level of *Nos2* and *Mrc1* in bone marrow–derived macrophage cultured with different tumor cell conditioned medium during in vitro macrophage polarization. Two-tail unpaired Student’s *t* test was used to calculate *P* value. (**F**) Frequency of *Nos2* expressing cells in total CD45 cells in different conditions. (**G**) Top differentially expressed genes in *Nos2* expressing myeloid cluster in PBS treated WT or KD tumors. (**H** and **I**) Association of *Nos2* ^+^ myeloid cluster gene signature *Z* score with patient clinical responses in IMvigor210 trial (CR = 15, PR = 27, SD = 35, and PD = 91) and Gide2019_PD1_melanoma cohort (*n* = 41). Data in (E) are presented as the means ± SEM. *P < 0.05, **P < 0.01, ***P < 0.001, and ****P < 0.0001. Significance was determined by two-tailed unpaired Student’s *t* test.

We next focused our analysis on macrophage, the most abundant immune populations in the TME (Fig. 4A). scRNAseq analysis of the macrophage transcriptomes in the KD and WT tumors showed that pathways including response to IFN-β, innate and humoral immune response, were enriched in macrophages from the KD tumors, suggesting a shift toward inflammatory status (Fig. 5A). In support of this observation, *Cd86* and *Cxcl9* expressions were up-regulated in proinflammatory macrophage clusters (clusters 0 and 1, *Cd86^+^*, *Cxcl10^+^*, *Cxcl9^+^*, and *Il1b^+^*) from KD tumors and were highest in KD tumors treated with anti–PD-1 (Fig. 5, B and C). Similarly, expression of genes associated with anti-inflammatory function, such as *Mrc1* and *Fn1*, were lower in the immunosuppressive macrophage clusters (*40*–*43*) (cluster 3, *Trem176^+^*; cluster 4, *Mrc1^+^*; cluster 7, *Arg1^+^*; cluster 13, *Folr2^+^*, *Trem176^+^*, and *Cd163^+^*; cluster 16, *Arg1^+^*; cluster 19, *Spp1^+^* and *Arg1^+^*; and cluster 21, *Fn1^+^*) from KD tumors and were lowest in KD tumors treated with anti–PD-1 (Fig. 5D). These data are consistent with recent studies suggesting a macrophage polarization role of Ptdss1 (*21*). In vitro macrophage polarization assay using tumor cell–conditioned medium provided further support of this notion, as shown by the increased *Nos2* expression and decreased *Mrc1* expression in bone marrow–derived macrophages cultured in KD conditioned media during M1 and M2 polarization process, respectively (Fig. 5E). Loss of PTDSS1 also increased the frequency of an iNOS^+^ (encoded by *Nos2*) myeloid cell cluster, which were typically observed in mice responding to ICT (*44*, *45*), and induction of this iNOS^+^ myeloid cell cluster by anti–PD-1 and PTDSS1 deficiency in tumor cells was synergistic (Fig. 5F). These cells in the KD tumors had lower expression of *Ccl24* and *Mrc1* (Fig. 5G), which are important for maintaining M2-like function (*46*). These cells also had higher expression of *Ccl5*, *Nos2*, and *AW112010* in KD tumors. Recent reports also suggest that *Ccl5* may have an antitumor role in the ICT setting (*47*), and this antitumor role may depend on CD4^+^ T cell infiltration (*48*). Together with the increased CD4^+^ T_eff_ frequency in KD tumors, these data suggest that knocking down *Ptdss1* may improve anti–PD-1 response via the *Ccl5*-CD4 T cell axis. *AW112010* is a noncoding RNA, which has pro-inflammatory function in bone marrow–derived macrophages and is essential for mucosal immunity (*49*). Recent study found that mutation in *AW112010* in macrophage cell line decreased the expression of pro-inflammatory gene *Il6* and increased the expression of anti-inflammatory gene *Il10* (*50*). The increased *AW112010* in the *Nos2* ^+^ myeloid population in KD tumors suggest that loss of Ptdss1 may polarize this myeloid population toward a pro-inflammatory state. A gene signature consisting of the top five up-regulated genes (*Ccl5*, *Apoe*, *Nos2*, *C1qb*, and *Ubb*) in the *Nos2* ^+^ myeloid population from the KD tumors correlated with clinical benefit for patients treated with anti–PD-1/PD-L1 (Fig. 5, H and I). Together, these results suggest that loss of *Ptdss1* in tumor cells shapes an inflammatory anti-TME.

### PTDSS1 amplification is common in cancer and predicts poor response to anti–PD-1 treatment

**Fig. 6.**
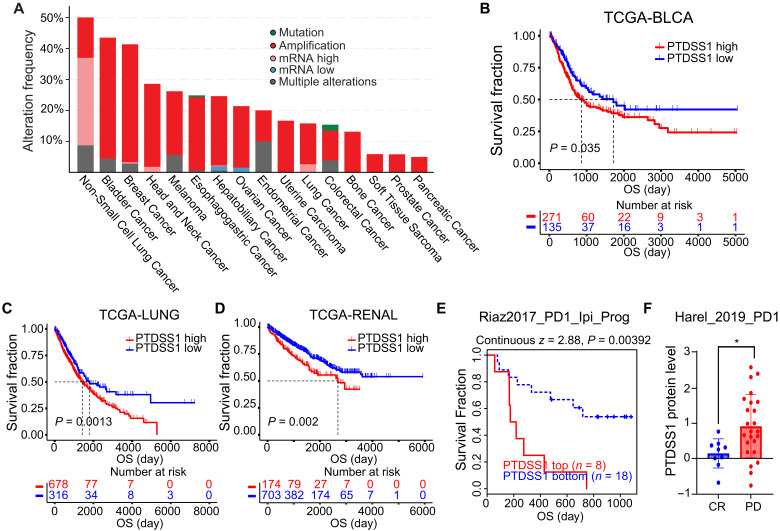
PTDSS1 level negatively correlated with cancer patient survival. (**A**) *PTDSS1* amplification status across tumor types profiled in TCGA Pan-cancer study. (**B** to **D**) Overall survival in TCGA bladder, lung, and renal cancer by *PTDSS1* expression level. (**E**) Correlation of *PTDSS1* RNA level with survival of patient who had previously been on anti–CTLA-4 therapy (ipilimumab, Ipi) and had progressed onto anti–PD-1 treatment in the Riaz_2017 cohort. (**F**) Correlation of PTDSS1 protein level with anti–PD-1 responses (CR, complete response; PD, progressive disease) in patients with melanoma treated with anti–PD-1 in the Harel_2019 cohort. Data in (F) are presented as the means ± SEM. *P < 0.05, **P < 0.01, ***P < 0.001, and ****P < 0.0001. Significance was determined by log-rank test for [(B) to (E)] and Mann-Whitney test for (F).

To extend our findings from mouse to human, we checked the amplification status and RNA level of *PTDSS1* in The Cancer Genome Atlas (TCGA). As shown in Fig. 6A, *PTDSS1* is frequently amplified (>20%) in a variety of cancer types, including bladder cancer, breast cancer, head and neck cancer, and melanoma (Fig. 6A). Correlation analysis also showed that *PTDSS1* expression is negatively correlated with cancer patient survival in various cancer types (Fig. 6, B to D), suggesting a protumor role. Moreover, analysis of the public available ICT datasets revealed that high *PTDSS1* RNA level and protein level in pretreatment human melanoma samples are correlated with poor patient survival (Fig. 6E) and are associated with poor response to anti–PD-1 treatment (Fig. 6F), respectively.

## DISCUSSION

In this study, we performed an in vivo CRISPR screen in a murine tumor model to systematically explore the tumor-specific genes involved in regulating responses to ICT and identified *Ptdss1*, which, when knocked out in cancer, can alter cancer cell fitness under selective pressure of anti–PD-1 therapy. We found that both genetic and pharmacological inhibition of *Ptdss1* in tumor cells significantly improved anti–PD-1 efficacy. Mechanistically, loss of *Ptdss1* in tumor cells enhanced immunogenicity, response to IFN-γ, and sensitivity to CD8^+^ T cell–mediated killing in a tumor cell–intrinsic manner. Moreover, *Ptdss1* loss promoted development of a pro-inflammatory TME consisting of an increased frequency of CD4^+^ T cells and iNOS^+^ myeloid cells in a tumor cell–extrinsic manner (fig. S3). Further, we demonstrated that a gene signature derived from these iNOS^+^ myeloid cells correlated with improved response to ICT in two different patient cohorts. Our results provide a strong rationale for combining *Ptdss1* inhibition and anti–PD-1 immunotherapy to improve outcomes for patients.

Here, we found that *Ptdss1* deficiency in tumor cells resulted in the up-regulation of genes involved in IFN response. Specifically, *Ptdss1*-deficient tumor cells were more responsive to IFN-γ stimulation through the STAT1 signaling pathway, resulting in greater MHC-I surface expression, and, ultimately, enhanced CD8^+^ T cell–mediated cell killing in vitro. This particular mechanism of action may explain why *Ptdss1* inhibition particularly synergizes with anti–PD-1 therapy. The mechanism by which *Ptdss1* inhibition or deficiency directly regulates the IFN response pathway will require additional studies. We found that PTDSS1 loss impairs RAS signaling, as shown by the enriched KRAS_SIGNALING_DN pathway (fig. S4A) and decreased pErk or pMEK levels (fig. S4, B and C) in KD cells. As KRAS has been shown to suppress STAT1 transcription (*51*), we hypothesize that PTDSS1 loss impairs RAS signaling, which in turn releases the suppression of STAT1 expression, enhancing tumor cell responsiveness to IFN-γ in a cell-intrinsic manner. While the primary reported role of *Ptdss1* is in the production of PS, there is discrepancy in the literature as to whether loss of *Ptdss1* results in substantial reduction of total PS species. For example, in *Ptdss1^−/−^* mice, while total serine-exchange activity in tissues was reduced by up to 85%, the PS content was unaltered in all tissue except for liver (*37*). This points to a potential unrecognized cell-intrinsic role of *Ptdss1* in regulating immunogenicity.

Our intratumor scRNAseq data revealed that *Ptdss1-*deficient tumor cells could profoundly remodel the immune microenvironment. We found that *Ptdss1-*deficient tumors correlated with the accumulation of iNOS^+^ myeloid cells that have been previously associated with ICT responsiveness (*44*, *45*). This population of iNOS^+^ cells was present both in *Ptdss1-*deficient tumors in the untreated setting as well as increased in response to anti–PD-1. Thus, we propose that the gene signature derived from this population could be used as a biomarker for patient selection and monitoring for anti–PD-1 treatment. Functional analyses confirmed that conditioned supernatant from *Ptdss1^KD^* tumor cells skewed macrophage polarization toward a more pro-inflammatory phenotype. However, further mechanistic studies are needed to elucidate the secreted factors directing this polarization. The combination of more immunogenic *Ptdss1^KD^* tumor cells and proinflammatory myeloid cells likely resulted in the increase of both effector CD8^+^ and T_H_1 CD4^+^ T cells, predisposing *Ptdss1^KD^* tumors to improved response to anti–PD-1 therapy.

Targeting PS has presented notable challenges in the clinical setting. While strategies directly targeting PS have shown antitumor activity and demonstrate synergize with ICT in various preclinical tumor models (*52*–*54*), negative results from a randomized phase 3 trial evaluating a PS monoclonal antibody in non–small cell lung suggest alternative approaches are necessary for targeting the PS pathway (*19*). One such strategy has been targeting various PS receptors such as MerTK (*55*). However, this could be limited by both PS receptor (*56*) and ligand redundancy (*57*). Here, we propose that directly targeting *Ptdss1* may overcome these limitations and improve response to ICT. It was previously reported that the PTDSS1 inhibitor, DS55980254, could induce endoplasmic reticulum stress and immunogenic cell death in *PTDSS2*-deficient tumors (*20*). However, *PTDSS2* is rarely mutated or deleted in patients with solid tumors, making the clinical utility of these findings difficult to interpret. We demonstrate that the combination of DS55980254 with anti–PD-1 therapy significantly improved tumor control and overall survival when compared to anti–PD-1 alone. Pairing this with our finding that *PTDSS1* is frequently amplified across diverse tumor types and associates with poor patient survival, these results indicates that *PTDSS1* represents a broadly relevant target for developing novel cancer therapeutic strategies.

In summary, we identified PTDSS1 as a regulator of tumor cell immunogenicity and antitumor immunity with both tumor cell–intrinsic and tumor cell–extrinsic roles. Given the frequent amplification of *PTDSS1* in patients with cancer and the negative correlation between *PTDSS1* level and response to ICT in some cancer types, our data suggest that combining PTDSS1 inhibition with ICT could serve as a promising therapeutic strategy for cancer treatment across tumor types.

## MATERIALS AND METHODS

### Animal experiments

All animal work was reviewed and approved (00000893-RN03) by MD Anderson Cancer Center’s Institutional Animal Care and Use Committee (IACUC). All mice were maintained in pathogen-free conditions. Four- to 6-week-old male C57BL/6 mice were purchased from the Charles River Laboratories. For CRISPR screen, 2 × 10^6^ cells from early passages of the cell pools were subcutaneously injected into each mouse to maintain 200× coverage of the library. Mice were then treated intraperitoneally with three doses of anti–PD-1 (BioXCell, BE0146) or PBS on days 3, 6, and 9 post-tumor inoculation (200/100/100 μg each in 100 μl of PBS). Tumor length and width was measured every 3 days using digital caliper. Tumor volume was calculated as (length × width^2^)/2. For tumor growth and survival experiment, 2 × 10^5^ KD or WT control cells were subcutaneously injected into mice, followed by the same treatment regime as the screen study. For in vivo proliferation assay, 2 × 10^5^ KD or control cells were subcutaneously injected into immune-deficient NSG (NOD.Cg-*Prkdc^scid^ Il2rg^tm1Wjl^*/SzJ) mice purchased from the Jackson Laboratory, followed by tumor growth monitoring. For PTDSS1 inhibitor study, 2 × 10^5^ parental MB49 cells were subcutaneously injected into mice, followed by treatment with either PBS, three doses of anti–PD-1, DS55980254 (10 mg/kg; ProbeChem, PC-49548) daily via oral gavage for a total of 14 days or anti–PD-1 plus DSS55980254. For scRNAseq experiment and tumor growth assays, 2 × 10^5^ KD or WT control cells were subcutaneously injected into mice, followed by the same treatment regime as the screen study, mice were euthanized on day 9 to collect tumor samples for scRNAseq.

### In vivo CRISPR screening and data analysis

Mouse bladder cancer cell line MB49 (Sigma-Aldrich, SCC148) was first transduced with Cas9-expression plasmid (Addgene 52962) followed by puromycin selection to make Cas9-expressing MB49 stable cell line. Mouse CRISPR knockout library was a gift from M. Bassik (Addgene, 1000000122). The library was divided into two subpools, each containing ~800 nontargeting control sgRNAs and ~10000 sgRNAs targeting around 1000 genes that encode kinases, phosphatases, and drug targets. MB49-Cas9 cells were transfected with viruses from the two subpools individually at an infection rate of 0.3 to create two cell pools. The cell pools were cultured in vitro for 5 days to ensure successful gene editing, followed by examination of sgRNAs representation by next-generation sequencing. Cell pools were transplanted subcutaneously into mice, followed by treatment with anti–PD-1 or PBS. Mice were euthanized on day 12 and tumor samples were collected. Genomic DNA was extracted from the tumor samples using a Quick-DNA Midiprep Plus kit (ZYMO Research). Barcoded sequencing libraries were prepared by polymerase chain reaction (PCR) as described (*58*). Pooled library was then sequenced on Illumina NextSeq500 Mid output platform by the Advanced Technology Genomics Core at The University of Texas MD Anderson Cancer Center. Software casTLE (*25*) was used for data analysis. In brief, sgRNA counts from each sample were first obtained by mapping the sequencing reads to the library reference, after sequencing depth normalization, sgRNA counts from the two subpools were combined and differential analysis was performed between tumor samples and original cell pools. sgRNA composition changes in the anti–PD-1–treated group and PBS treated group were compared against each other to determine anti–PD-1–specific changes. Functional enrichment analysis was performed using Metascape (https://metascape.org) (*59*).

### Cell culture and generation of knockdown clones

Cell lines were cultured in high-glucose Dulbecco’s Modified Eagle Medium supplemented with 10% fetal bovine serum (FBS) and penicillin-streptomycin (100 U/ml). *Ptdss1* KD (KD) and control cell (WT) cells were established by lentiviral infection of parental cells using pMD2.G and psPAX2 lentiviral packaging system and LentiCRISPRv2GFP (Addgene, 82416) with or without sgRNAs targeting *Ptdss1*. sgRNA sequences information is included in table S2.

### In vitro proliferation assay

MB49 cells (1 × 10^4^) were plated in 96-well plate in triplicates for each cell line. Cells were then cultured for 5 days in IncuCyte (Sartorius). Images were taken every 6 hours to measure cell confluency. Cell proliferation data were generated using the IncuCyte software ZOOM.

### Western blotting

Whole-cell lysates were prepared on ice in RIPA buffer (Pierce) with protease and phosphatase inhibitors (Biomake). Protein concentration was measured by Pierce BCA Protein Assay kit (Thermo Fisher Scientific). Twenty micrograms of protein from each sample was loaded on 4 to 20% precast gradient gel (Bio-rad), transferred to polyvinylidene difluoride membranes and subjected to staining with the following antibodies: Cas9 (Cell Signaling Technology, 65832), β-actin (Cell Signaling Technology, 4970S), Stat1 (Cell Signaling Technology, 9172S), and pStat1(Tyr^701^) (Cell Signaling Technology, 9167S). Images were taken on ChemiDoc MP system (Bio-rad).

### RNA isolation and real-time quantitative PCR

Total RNA was extracted from cells using Quick-RNA miniprep kit (ZYMO Research). cDNA was synthesized using 5X All-In-One RT mastermix (Applied Biological Materials Inc.). Real-time quantitative PCR (qPCR) was performed with BrightGreen 2X qPCR MasterMix (Applied Biological Materials Inc.) on the 7500 Fast Real-Time PCR System (Applied Biosystems). Samples were run in triplicate and normalized to *Gapdh*. Relative mRNA expression level was determined using the 2−ΔΔCt method.

### RNA sequencing and data analysis

Total RNA samples were isolated as described above. Stranded mRNA-seq was performed on NextSeq500 high-output platform by the Advanced Technology Genomics Core at The University of Texas MD Anderson Cancer Center. Sequencing reads were aligned to mouse genome mm10 using HISAT2 software, transcripts were quantitated using StringTie. Data normalization and differential expression analysis were performed with R package DESeq2. GSEA was performed using GSEA Java package (version 4.0) and hallmark gene sets from MSigDB (version 7.4).

### Flow cytometry

Cells were trypsinized, washed with fluorescence-activated cell sorting (FACS) buffer (PBS + 5% FBS + 2 mM EDTA), and incubated with TruStain FcX anti-mouse CD16/32 antibody (BioLegend, 101319) for 10 min, followed by staining with the following fluorochrome-conjugated antibodies on ice for 30 min: H-2Kb/H-2Db (BioLegend, 116520) and β2-microglobulin (BD Biosciences, 745291). Cells were analyzed on LSR II cytometer (Becton Dickinson).

### Antigen presentation assay and in vitro T cell killing assay

For antigen presentation assay, KD or WT tumor cells were first pulsed with OVA^257–264^ peptide SIINFEKL (100 ng/ml; InvivoGen) for 6 hours, followed by flow cytometry analysis of SIINFEKL bounded H2Kb level (Miltenyi Biotec, 130-102-175). For T cell killing assay, CD8^+^ T cells were first isolated from spleen of male OT-1 mouse using CD8^+^ T cell isolation kit (STEMCELL Technologies), and subsequently cultured in T cell culture media supplemented with mouse interleukin-2 (IL-2; 30 U/ml; PeproTech), 50 μM β-mercaptoethanol (Gibco), and CD3/CD28 magnetic beads (Gibco) for 5 days. On the day of tumor cell–T cell coculture, tumor cells were pulsed with SIINFEKL for 2 hours, then cell culture media was removed, activated T cells were added to tumor cell culture at different ratios and cultured for 24 hours. Killing of tumor cell by T cell was measured by flow cytometry analysis of 7-AAD staining (eBioscience).

### scRNAseq and data analysis

Tumors were first minced with scissors and digested with Liberase TL (0.67 mg/ml; Sigma-Aldrich) and deoxyribonuclease (0.4 mg/ml; Sigma-Aldrich) for 30 min at 37°C with continuous rotation. Samples were further homogenized on 70-μm cell strainer placed on ice to get single-cell suspension. CD45^+^ cells were isolated with CD45 microbeads (Miltenyi Biotec). scRNAseq library was generated using the Chromium Single Cell 3′ GEM Library & Gel Bead Kit (10X Genomics, PN-1000075) and the 10X Genomics Chromium Controller instrument. In brief, 10,000 cells were loaded to the controller to generate single-cell gel beads emulsions. Reverse transcription, cDNA amplification, and sample indexing were then performed to generate barcoded single-cell libraries. Pooled library was then sequenced on Novaseq 6000 high-output platform by the Advanced Technology Genomics Core at The University of Texas MD Anderson Cancer Center. Sequencing data were first processed using the Cell Ranger software from 10X Genomics to obtain raw read counts. R package Seurat was used to filter for high-quality singlets, integrate samples from different condition, identify clusters, and analyze cluster frequencies and gene expression following analysis. Gene signature *z* score was calculated as follows: *z* score for each gene is calculated, added up, then divided by square root of number of genes.

### Macrophage polarization assay

Bone marrow cells from male WT C57BL/6 mice were first collected, followed by treatment of macrophage colony-stimulating factor (20 ng/ml; Miltenyi Biotec) for 7 days to enable macrophage differentiation. Bone marrow–derived macrophages were then cultured in WT or KD MB49 cell conditioned medium supplemented with either IFN-γ (20 ng/ml) and lipopolysaccharide (100 ng/ml) for M1 polarization or IL-4 (10 ng/ml) for M2 polarization. The next day, RNA was extracted from macrophages and real-time qPCR was performed to quantify *Nos2* and *Mrc1* expression levels.

### Statistical analysis

All experiments, except for sequencing experiments, were repeated at least three times to ensure data reproducibility. Two-way analysis of variance (ANOVA), Kaplan-Meier survival analysis, two-tail unpaired Student’s *t* test, and Mann-Whitney test were performed in GraphPad Prism 9. Sample sizes for each experiment are indicated in figure legends. Data are expressed as mean with SEM. *P* values lower than 0.05 were considered statistically significant. *P < 0.05, **P < 0.01, ***P < 0.001, and ****P < 0.0001; ns (not significant, *P* > 0.05).
