# Supplementary material for: Loss of PTDSS1 in tumor cells improves immunogenicity and response to anti–PD-1 therapy
Source: Sci Adv. 2025 Sep 10;11(37):eadx8134. doi: 10.1126/sciadv.adx8134 (PMC12422196; doi:10.1126/sciadv.adx8134)
Supplement: Supplementary file 1 — Figs. S1 to S4 Tables S1 and S2 [file sciadv.adx8134_sm.pdf]

Science Advances
AAAS

Supplementary Materials for

Loss of PTDSS1 in tumor cells improves immunogenicity and response to anti–PD-1 therapy

Jielin Liu *et al.*

Corresponding author: Padmanee Sharma, padsharma@mdanderson.org

Sci. Adv. **11**, eadx8134 (2025)
DOI: 10.1126/sciadv.adx8134

This PDF file includes:

Figs. S1 to S4
Tables S1 and S2

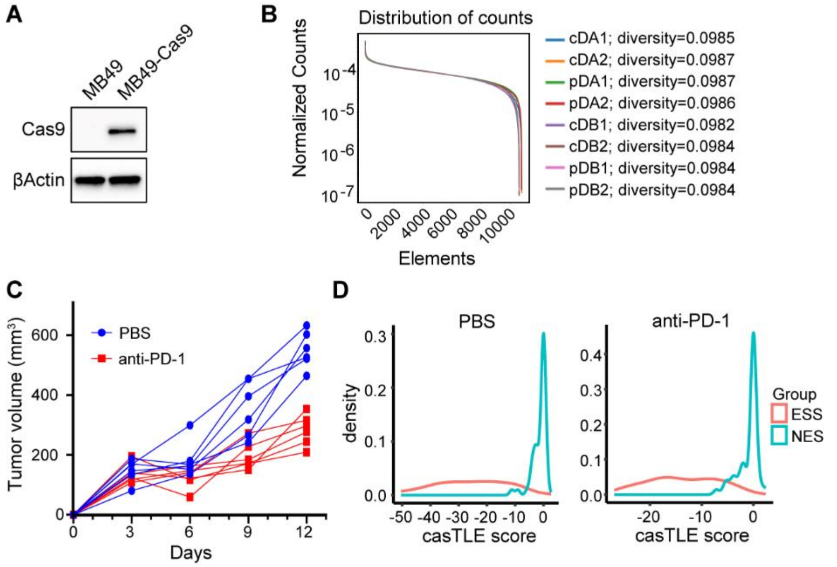

Supplementary Fig. 1. In vivo CRISPR screen performance.

(A) Western blot analysis of Cas9 level in MB49 Cas9 expressing cells. **(B)** sgRNA library representation in transfection plasmid libraries (p) and lentivirus transfected cell libraries (c). **(C)** Tumor growth curve of the mice in CRISPR screen (n=6 for each treatment condition). **(D)** Screening quality represented by distributions of sgRNAs targeting essential genes (ESS) and non-essential genes (NES).

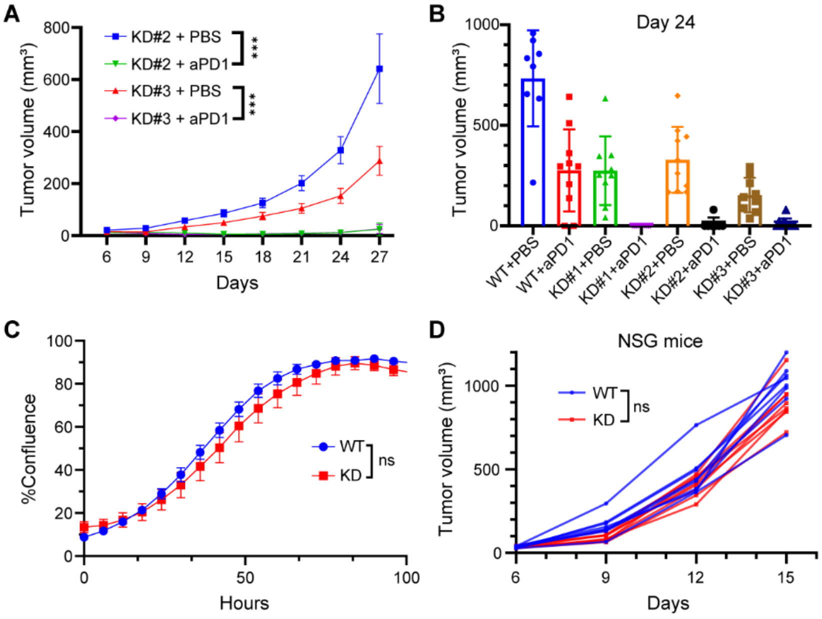

Supplementary Fig. 2. Ptdss1 deficiency slows down tumor growth when combined with anti-PD-1.

(A) Tumor growth curves of mice transplanted with KD#2 and KD#3 MB49 cells treated with PBS or anti-PD-1. **(B)** Representative tumor size of WT or KD tumors treated with PBS or anti-PD-1 on day 24. **(C)** Incucyte cell proliferation assay results of WT and KD clones. P value was calculated by Mann Whitney test. **(D)** Individual tumor growth curve of NSG mice transplanted with control or KD cells (n=10 for each group). Data are presented as the means ± SEM. ***P < 0.001; ns, not significant. Significance was determined by two-way ANOVA for (A)(C)(D).

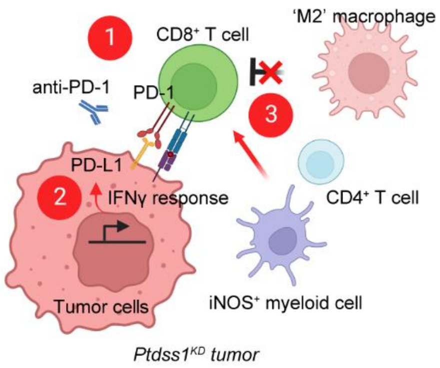

Supplementary Fig. 3. Working model for Ptdss1 deficiency combined with anti-PD1 combination therapy.

Inhibition of Ptdss1 in tumor cells not only increases tumor cells response to IFNγ and immunogenicity, but also induce changes in the tumor microenvironment, favoring development of an inflammatory anti-tumor TME, thus synergizing with anti-PD1 in promoting tumor clearance. Created in BioRender. Liu, J. (2025) https://BioRender.com/mq3egsr.

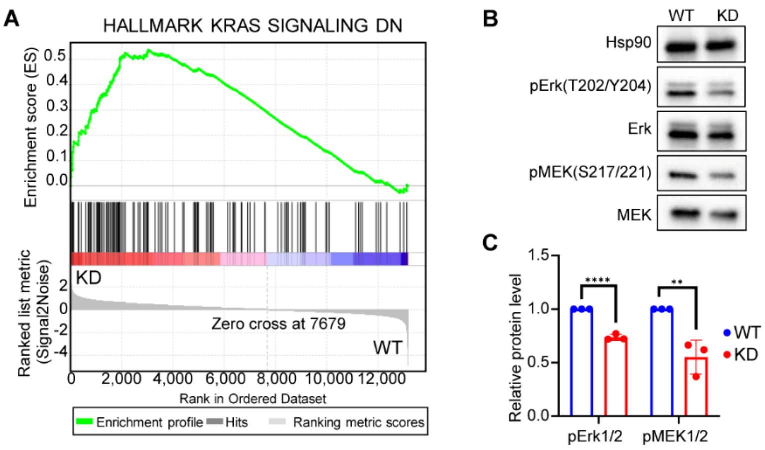

Supplementary Fig. 4. Loss of Ptdss1 downregulates Ras signaling.

(A) Gene set enrichment analysis result showing KRAS_SIGNALING_DN gene set was enriched in KD cells. **(B,C)** Western blot results and quantification of pErk1/2 and pMEK1/2 levels in WT

and KD cells. Data are presented as the means ± SEM. $^{**}P < 0.01$; $^{****}P < 0.0001$. Significance was determined by two-tailed unpaired Student's t-test for (C).

Table S1. *In vivo* CRISPR knockout screen results (anti-PD-1 specific)

Symbol	casTLE.Effect.x	casTLE.Score.x	casTLE.p.value.x	casTLE.Effect.y	casTLE.Score.y	casTLE.p.value.y	casTLE.Score.x.signed	casTLE.Score.y.signed
H2-K1	3.4	12.6	0.0168	4.6	47.6	0.00001	12.6	47.6
B2M	2.6	10.8	0.0298	3.5	33.9	0.00001	10.8	33.9
MELK	-5.1	5.85	0.137	7.7	21.4	0.00019	-5.85	21.4
H2-Q7	-1.3	0.988	0.595	5.2	18.2	0.00081	-0.988	18.2
HSPB8	-0.2	0.0512	0.876	6.8	17.6	0.00084	-0.0512	17.6
HADH	4	1.72	0.475	7.1	15.7	0.0013	1.72	15.7
SLC7A2	0	0	0.925	6.1	13.6	0.00232	0	13.6
SERPINC1	-1.3	1.3	0.538	6.9	13	0.00292	-1.3	13
MAPK4	2.2	5.18	0.168	6.9	12.9	0.00313	5.18	12.9
TRIB1	4.3	2.66	0.369	4.3	9.89	0.00774	2.66	9.89
DGKE	-2.4	3.54	0.282	-6.8	9.31	0.00989	-3.54	-9.31
CKS2	-5.1	4.69	0.195	-5.8	9.4	0.00941	-4.69	-9.4
SOAT2	-0.7	0.383	0.745	-5.6	9.43	0.00933	-0.383	-9.43
CDK4	-1.6	1.38	0.525	-5.9	9.48	0.00916	-1.38	-9.48
PCBD1	4.6	0.386	0.744	-5.5	9.54	0.00897	0.386	-9.54
ALLC	-4.7	9.32	0.0471	-5.4	9.67	0.00845	-9.32	-9.67
IKBKB	-5.2	8.75	0.0563	-6.7	9.68	0.00841	-8.75	-9.68
EFNB2	-1.5	0.824	0.63	-6.3	10.3	0.00655	-0.824	-10.3
PIKFYVE	-3.7	1.41	0.52	-6.2	10.3	0.00655	-1.41	-10.3
PTDSS1	-5.7	13.2	0.0139	-6.5	10.7	0.00558	-13.2	-10.7
EPHA1	-1.2	0.505	0.709	-5.2	10.7	0.00558	-0.505	-10.7
MTMR9	-3	13.8	0.0119	-5.8	10.9	0.00523	-13.8	-10.9
CSNK2A1	-7	1.18	0.559	-5.8	10.9	0.00523	-1.18	-10.9
ATP5O	-5.2	12.4	0.0179	-5.9	11.1	0.00479	-12.4	-11.1
CTSG	-0.3	0.108	0.846	-5.7	11.3	0.00457	-0.108	-11.3
HMOX2	-4.9	3.66	0.271	-6.3	11.5	0.00437	-3.66	-11.5
CTDSP2	-3.1	5.28	0.163	-7.1	11.6	0.00427	-5.28	-11.6
ARPC2	-2.9	13.5	0.0129	-3.7	11.7	0.00414	-13.5	-11.7
CDK11B	-2.1	2.1	0.428	-4.9	11.8	0.00405	-2.1	-11.8
TRAPPC1	-3.9	8.83	0.0551	-4.9	11.9	0.004	-8.83	-11.9
PIK3C2A	-3.4	6.17	0.124	-6.5	12.3	0.00371	-6.17	-12.3

MAN2A1	-0.4	0.0182	0.901	-5.1	12.6	0.00345	-0.0182	-12.6
GSTA3	-7.1	0.744	0.648	-4.8	13	0.00292	-0.744	-13
PIK3C2B	-4.5	7.39	0.087	-5.7	13.2	0.0027	-7.39	-13.2
CHKA	-2.2	4.58	0.202	-6.5	13.6	0.00232	-4.58	-13.6
PRKAA1	-5.5	3.65	0.272	-6.3	13.8	0.00228	-3.65	-13.8
RIOK1	-6.1	14.3	0.0103	-6.4	14.2	0.00212	-14.3	-14.2
ROS1	-5.1	9.23	0.0484	-6.2	15.7	0.0013	-9.23	-15.7
RAB7	-3.8	4.95	0.18	-6.7	15.7	0.0013	-4.95	-15.7
2810408M09RIK	-3.6	8.16	0.0678	-4.6	16.8	0.00092	-8.16	-16.8
TRAPPC4	-2.4	3.02	0.332	-4.9	18.1	0.00081	-3.02	-18.1
EIF3B	-2.9	7.97	0.0722	-5.9	18.3	0.0008	-7.97	-18.3
MED1	-3.7	4.32	0.219	-6	18.8	0.00077	-4.32	-18.8
PPP1R10	-3.5	13.8	0.0119	-4.5	18.9	0.00077	-13.8	-18.9
PKMYT1	-3.7	11.6	0.0235	-6.4	20.6	0.00028	-11.6	-20.6
LSM6	-5.2	7.42	0.0861	-5.3	21.4	0.00019	-7.42	-21.4
NDUFB10	-4.1	12	0.0205	-6.4	21.6	0.00016	-12	-21.6
CAML	-3.9	8.74	0.0565	-6.2	23.6	0.00005	-8.74	-23.6

In vivo CRISPR screen results showing the differential enriched or depleted sgRNAs in anti-PD-1 treated group. casTLE.p.value.y < 0.01 was used as cutoff to determine significant enrichment (casTLE.Score.y.signed > 0) and depletion (casTLE.Score.y.signed < 0) in anti-PD-1 treated tumors.

Table S2. Primer sequences

	Forward (5'-3')	Reverse (5'-3')
sgRNA primers		
Ptdss1-sgRNA#1	CACCGTCGTGTAGCATATCGAAGAT	AAACATCTTCGATATGCTACACGAC
Ptdss1-sgRNA#2	CACCGTCGATATGCTACACGAGAAG	AAACCTTCTCGTGTAGCATATCGAC
Ptdss1-sgRNA#3	CACCGACCAAAAACCATTCGCCATA	AAACTATGGCGAATGGTTTTTGGTC
**qPCR primers**		
Gapdh	AACAGCAACTCCCACTCTTC	CCTGTTGCTGTAGCCGTATT
Ptdss1	GCAGGACTCTGAGCAAGGATG	GGCGAAGTACATGAGGCTGAT
**CRISPR screen primers**		
oMCB_F1_1562	AGGCTTGGATTTCTATAACTTCGTATAGCATACATTATAC	
oMCB_R1_1563	ACATGCATGGCGGTAATACGGTTATC	
oMCB_F2_1439	CAAGCAGAAGACGGCATACGAGATGCACAAAAGGAAACTCACCCT	
oMCB_Seq_1672	GCCACTTTTTCAAGTTGATAACGGACTAGCCTTATTTAAACTTGCTATGCTGTTTCCAGCTTAGCTCTTAAAC	
oMCB_R2_Index	AATGATACGGCGACCACCGAGATCTACACGATCGGAAGAGCACACGTCTGAACTCCAGTCACXXXXXXCGACTCGGTGCCACTTTTTC	
